# Supplementary material for: 1T-MoS2 Coordinated Bimetal Atoms as Active Centers to Facilitate Hydrogen Generation
Source: Materials (Basel). 2021 Jul 22;14(15):4073. doi: 10.3390/ma14154073 (PMC8347348; doi:10.3390/ma14154073)
Supplement: Supplementary file 1 [file materials-14-04073-s001.zip › materials-1281192-supplementary.pdf]

materials

Supplementary materials

1T-MoS_2 Coordinated Bimetal Atoms as Active Centers to Facilitate Hydrogen Generation

Qiong Peng *, Xiaosi Qi *, Xiu Gong and Yanli Chen

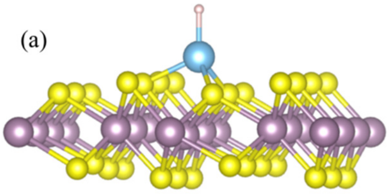

Figure S1. Two different adsorption sites of H atom on the Ti@1T-MoS_2 surface. (a) H adsorption on the Ti atom; (b) H adsorption on the S atom.

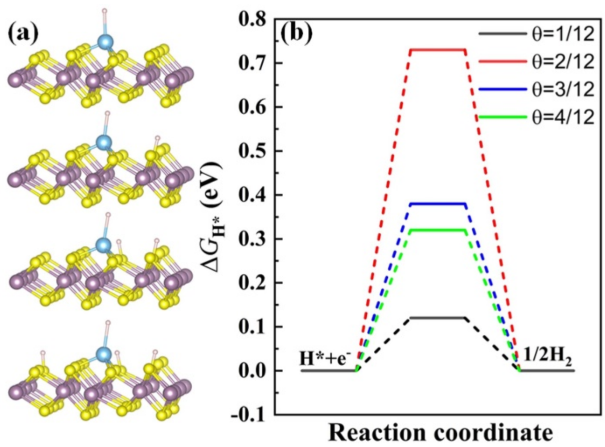

Figure S2. (**a**) Side views of the lowest energy structure and (**b**) corresponding Gibbs free energy of H* adsorption (ΔG_{H^*}) at different H coverage on the Ti@1T-MoS_2 surface.

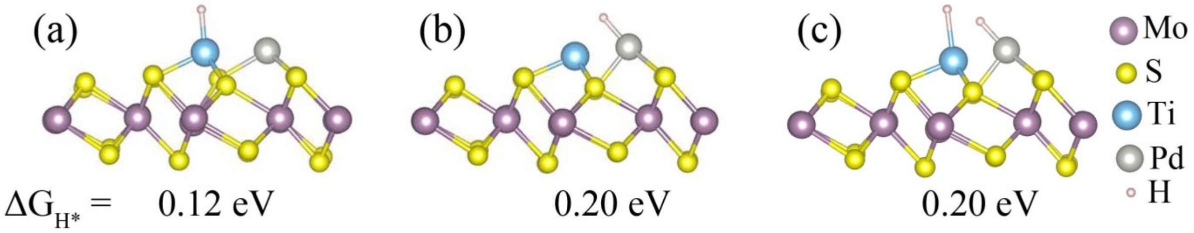

Figure S3. One H atom adsorption on (**a**) the Ti and (**b**) Pd site of PdTi@1T-MoS_2 catalyst, where the H atom is inclined to be directly bonded to the Ti atom with lower H* adsorption free energy ΔG_{H^*}. (**c**) The second H atom adsorption on the Pd site.

Table S1. The elastic constants C_{11}, C_{22}, C_{12}, C_{66} (N/m) used to evaluate whether the TM@1T-MoS_2 systems meet the mechanical stability criteria, namely, $C_{11} > 0$, $C_{66} > 0$ and $C_{11}×C_{22} > C_{12}×C_{12}$.

System	C_{11}	C_{22}	C_{12}	C_{66}	Meeting Criteria
graphene	357.0	357.0	63.0	147.0	yes
Graphene [1]	352.0	352.0	62.6	144.7	yes
2H-MoS_2	135.9	135.9	33.3	51.3	yes
2H-MoS_2 [2]	128.4	128.4	32.6	47.9	yes
1T-MoS_2	96.5	96.5	-7.5	52.0	yes
Ti@1T-MoS_2	86.7	63.3	46.8	9.1	yes
V@1T-MoS_2	78.2	94.0	21.8	1.4	yes
Co@1T-MoS_2	114.5	102.5	14.4	9.6	yes
Ni@1T-MoS_2	116.1	108.1	13.7	10.2	yes
Cu@1T-MoS_2	119.0	127.8	16.1	10.4	yes
Zr@1T-MoS_2	108.3	−80.7	112.3	9.1	no
Nb@1T-MoS_2	77.7	96.1	25.1	−3.4	no
Rh@1T-MoS_2	114.8	106.2	14.9	9.3	yes
Pd@1T-MoS_2	111.6	117.8	16.5	10.0	yes
Ag@1T-MoS_2	116.7	126.4	17.2	10.2	yes
Hf@1T-MoS_2	95.2	88.3	32.5	8.1	yes

Table S2. The total energy E_{tot} and free energy ΔG_{H^*} of H* adsorption on site (a) and (b) shown in Figure S1, as well as the overpotential η_{HER} (V). The bold font marks the more negative E_{tot}, corresponding to the energetically most favorable configurations of H* adsorption.

System	E_{tot} (eV)		ΔG_{H^*} (eV)		η_{HER} (V)
	site-a	site-b	site-a	site-b	
Ti@1T-MoS_2	**−273.28**	−272.10	0.12	1.31	0.12
V@1T-MoS_2	**−273.53**	−272.45	0.13	1.21	0.13
Co@1T-MoS_2	−270.74	**−270.76**	0.44	0.41	0.41
Ni@1T-MoS_2	**−269.45**	−269.31	0.58	0.72	0.58
Cu@1T-MoS_2	−267.51	**−267.55**	0.81	0.77	0.77
Rh@1T-MoS_2	**−270.86**	−270.55	0.42	0.73	0.42
Pd@1T-MoS_2	−269.06	**−269.23**	0.53	0.36	0.36
Pd@1T-MoS_2[3]	-	**-**	0.49	0.35	0.35
Ag@1T-MoS_2	−266.36	**−266.66**	0.92	0.62	0.62
Hf@1T−MoS_2	**−276.15**	−274.26	−0.46	1.42	0.46

Table S3. Total energy E_{tot} and free energy ΔG_{H^*} of H* adsorbed on different sites shown in Figure 5b for bimetal atoms immobilized on the 1T−MoS_2 surface. The bold font marks the energetically most favorable configurations of H* adsorption.

System	E_{tot} (eV)				ΔG_{H^*} (eV)	
	I	II	III	IV	I	II
TiTi@1T−MoS_2	**−281.09**	−281.06	−279.42	−279.38	−0.62	−0.59
VTi@1T−MoS_2	**−280.99**	−280.46	−280.19	−279.79	0.00	0.53
NiTi@1T−MoS_2	**−277.80**	−277.57	−277.41	−276.98	0.19	0.43
CuTi@1T−MoS_2	**−276.36**	−275.73	−275.36	−275.13	0.01	0.64
PdTi@1T−MoS_2	**−277.33**	−277.26	−277.05	−276.54	0.12	0.20

Table S4. H adsorption Gibbs free energy ΔG_{H*} of $TM_{Sv}Ti@1T{-}MoS_2$ (TM = Ti, V, Ni, Cu, Pd and Pt) at H coverage θ = 1/12 (one H adsorption) and 2/12 (two H adsorption), where Ti atom was coordinated with three S atoms (site−S) and the other TM was deposited into the S vacancy (site−Sv).

System	ΔG_{H*} (θ = 1/12)		ΔG_{H*} (θ = 2/12)
	site−S	site−Sv	
$Ti_{Sv}Ti@1T{-}MoS_2$	**0.08**	0.45	0.63
$V_{Sv}Ti@1T{-}MoS_2$	0.18	**0.15**	0.34
$Ni_{Sv}Ti@1T{-}MoS_2$	0.64	**−0.36**	0.40
$Cu_{Sv}Ti@1T{-}MoS_2$	0.24	**−0.07**	0.36
$Pd_{Sv}Ti@1T{-}MoS_2$	0.35	**0.09**	−0.05
$Pt_{Sv}Ti@1T{-}MoS_2$	0.37	**0.28**	0.05

References

1. Peng, Q.; Han, L.; Lian, J.; Wen, X.; Liu, S.; Chen, Z.; Koratkar, N.; De, S., Mechanical degradation of graphene by epoxidation: Insights from first−principles calculations. *Phys. Chem. Chem. Phys.* **2015,** *17*, 19484–19490.
2. Peng, Q.; De, S., Outstanding mechanical properties of monolayer MoS_2 and its application in elastic energy storage. *Phys. Chem. Chem. Phys.* **2013,** *15*, 19427–19437.
3. Lau, T. H. M.; Wu, S.; Kato, R.; Wu, T.−S.; Kulhavý, J.; Mo, J.; Zheng, J.; Foord, J. S.; Soo, Y.−L.; Suenaga, K.; et al. Engineering monolayer 1T−MoS_2 into a bifunctional electrocatalyst via sonochemical doping of isolated transition metal atoms. *ACS Catal.* **2019,** *9*, 7527–7534.
